# Postbiotics-parabiotics: the new horizons in microbial biotherapy and functional foods

**DOI:** 10.1186/s12934-020-01426-w

**Published:** 2020-08-20

**Authors:** Basavaprabhu H. Nataraj, Syed Azmal Ali, Pradip V. Behare, Hariom Yadav

**Affiliations:** 1 Technofunctional Starters Lab, National Collection of Dairy Cultures (NCDC), Dairy Microbiology Division, ICAR-National Dairy Research Institute, Karnal, Haryana 132001 India; 2 Proteomics and Cell Biology Lab, Animal Biotechnology Center, ICAR-National Dairy Research Institute, Karnal, Haryana 132001 India; 3 Department of Internal Medicine-Molecular Medicine and Microbiology and Immunology, Wake Forest School of Medicine, Biotech Place, Room 2E-034, 575 North Patterson Ave, Winston-Salem, NC 27101 USA

**Keywords:** Probiotics, Postbiotics, Paraprobiotics, Functional foods, Health benefits

## Abstract

Probiotics have several health benefits by modulating gut microbiome; however, techno-functional limitations such as viability controls have hampered their full potential applications in the food and pharmaceutical sectors. Therefore, the focus is gradually shifting from viable probiotic bacteria towards non-viable paraprobiotics and/or probiotics derived biomolecules, so-called postbiotics. Paraprobiotics and postbiotics are the emerging concepts in the functional foods field because they impart an array of health-promoting properties. Although, these terms are not well defined, however, for time being these terms have been defined as here. The postbiotics are the complex mixture of metabolic products secreted by probiotics in cell-free supernatants such as enzymes, secreted proteins, short chain fatty acids, vitamins, secreted biosurfactants, amino acids, peptides, organic acids, etc. While, the paraprobiotics are the inactivated microbial cells of probiotics (intact or ruptured containing cell components such as peptidoglycans, teichoic acids, surface proteins, etc.) or crude cell extracts (i.e. with complex chemical composition)”. However, in many instances postbiotics have been used for whole category of postbiotics and parabiotics. These elicit several advantages over probiotics like; (i) availability in their pure form, (ii) ease in production and storage, (iii) availability of production process for industrial-scale-up, (iv) specific mechanism of action, (v) better accessibility of Microbes Associated Molecular Pattern (MAMP) during recognition and interaction with Pattern Recognition Receptors (PRR) and (vi) more likely to trigger only the targeted responses by specific ligand-receptor interactions. The current review comprehensively summarizes and discussed various methodologies implied to extract, purify, and identification of paraprobiotic and postbiotic compounds and their potential health benefits.

## Introduction

Food is a paramount basic need of life that clinches the nutritional requirement of an individual. The nutrients like fats, carbohydrates, and proteins pledge energy for growth and maintenance, whereas non-nutrient factors (fiber, phytochemicals, antioxidants, vitamins, minerals, probiotics, prebiotics, etc.) augment human health by positively modulating the host physiology and global epigenetic imprints [1–4]. Dietary intake of selected categories of foods comprising active components regulate the disease controlling mechanisms either as prophylactics or therapeutics, and such foods are typically called as nutraceuticals or foodiceuticals or functional foods or medifoods [5]. An intense innovation in the field of functional food has pawed way to generate an extensive range of health-promoting bioactive compounds such as probiotics, prebiotics, phytochemicals or herbs, natural antioxidants, bioactive peptides, etc. [6]. The manifestations of these active biologicals naturally in food or external fortification flag the food functional [7, 8]. Japan is the first country to propose legislation for the specific regulatory approval procedures of functional foods that were implemented as Food for Specific Health Uses (FOSHU) [9, 10]. Thereafter, several other countries have also structured their regulatory enforcement actions and civil litigations to govern the regulatory issues regarding functional foods such as US Federal Food and Drug Administration in the United States (USA), Food Safety and Standards Authority of India (FSSAI) in India, China Food and Drug Administration (CFDA) in China, European Union (EU) in Europe, National Sanitary Surveillance Agency (NSSA) in Brazil, etc. [11]. The functional foods can be defined as “any food that has a positive impact on an individual’s health, physical performance, or state of mind, in addition to its nutritious value” [9]. Also, it should serve to regulate a particular body process, such as enhancement of biological defense mechanisms, prevention of specific diseases, control of physical and mental disorders, and slowing of the aging process [9]. However, these functional foods can be further categorized as natural, transformed, fortified, and enhanced foods [9]. The rapid industrialization and modernization coupled with plummeting in the rate of consumption of health-promoting natural foods have witnessed the emergence of different health complications at an early age of human life [12–14]. Fascinatingly, consumer awareness and acceptance of functional foods to counteract lifestyle diseases have been recently amplified. This consumer interest is indeed driving the global functional food sector with an economic momentum of more than US$180 billion with the global annual demand for functional foods has anticipated rising at 8% [15].

Probiotics are among the amply studied and applied functional food ingredients. Probiotics are defined as “live microorganisms that, when administered in adequate amounts, confer a health benefit on the host” [16]. *Lactobacillus* and *Bifidobacterium* are the most studied probiotic genera. However, *Bacteroides* and *Clostridium* genera are emerging as next-generation probiotics irrespective of their safety issues [17]. To address such issues, the European Food Safety Authority (EFSA) has granted the Qualified Presumption of Safety (QPS) status to only a total of 32 *Lactobacillus* species for human applications considering their safety perspectives [18]. Hitherto, probiotics have been investigated for their ability to surpass gut functioning, alleviation of lactose intolerance, enhancement of immune function, anti-carcinogenic, anti-diabetic, anti-oxidative, anti-aging, antimicrobial, and anti-biofilm actions [19, 20].

Despite several health benefits, investigations on probiotics have highlighted few limitations such as unknown molecular mechanisms, strain-specific behaviors, short-lived, niche-specific action of probiotics (allochthonous or autochthonous), developing antibiotic resistance, virulence genes transfer, ambiguous beneficial effects, issues about the maintenance of viability and stability in the production process, a hindrance for colonization of commensal gut microflora, ability to cause opportunistic infections, inflammatory response infective endocarditis, sepsis, bacterial translocation to tissue or blood, and bacteremia in immunocompromised individuals are significant bottlenecks [21–23]. The low concentrations of probiotic derived biologically active compounds found in specific target sites in the course of traditional application of live probiotic microorganisms (live biotherapeutics) were found ineffective at in vivo conditions [24, 25]. On the other note, live probiotics have been reported to be affected by various host-specific factors in the gastrointestinal tract (GIT) that subsequently activate several bacterial genes for degradation and production of different nutrients by various metabolic pathways [26, 27]. To address such issues, postbiotic components derived from probiotics are probably favorable and promising alternative supplements for human health and wellness thereof.

## Concepts and definition

Several investigators have proposed different terminologies to describe postbiotics and paraprobiotics such as non-viable probiotics, inactivated probiotics, non-biotics, ghost probiotics, and metabiotics [24, 28, 29]. Of note, paraprobiotics have been defined alike the Food and Agriculture Organization/World Health Organization (FAO/WHO) definition of probiotics with minor modifications as “inactivated (non-viable) microbial cells, which, when administered in sufficient amounts, confer benefits to consumers” [30].

However, the verbal inconsistencies in defining postbiotics were streamlined by a recent opinion article [31]. Accordingly, (i) POSTBIOTICS may be defined as “non-viable bacterial products or metabolic products from microorganisms that have biological activity in the host (ii) PARAPROBIOTICS (also called ghost or inactivated probiotics) that are “non-viable microbial cells (either intact or broken) or crude cell extracts which when administered (either orally or topically) in adequate amounts, confer a benefit on the human or animal consumer”; and (iii) PROBIOCEUTICALS/PROBIOTACEUTICALS which defines probiotic derived factors such as reuterin from *Lactobacillus reuteri*.

Most importantly, the latest scientific literature has highlighted the widely accepted definition of paraprobiotics/ghosh probiotics as “the inactivated/dead/non-viable microbial cells of probiotics (intact or ruptured containing probiotic cell components upon lysis) or crude cell extracts (i.e. with complex chemical composition)” [32]. By contrast, postbiotics are the complex mixture of healthy metabolic products or secreted components of probiotics in cell-free supernatants such as enzymes, secreted proteins, short chain fatty acids, vitamins, amino acids, peptides, organic acids, etc. [33, 34]. Although the tentative term postbiotics has been widely used so far, hitherto there is no definition recommended by international regulatory bodies or scientific associations.

Since the specific action of postbiotics relies on definite dosage levels, most studies have failed to fix a specific dose of postbiotics/paraprobiotics to ensure the beneficial effects alike probiotics at 10^9^ viable cells. To investigate the effectiveness of postbiotics, currently, there have been a handful of comparative studies conducted at the in vitro and in vivo levels, and such studies suggest the similar potentialities of postbiotics over the probiotics in terms of demonstrating various health benefits on the host [35–37]. Singh et al. have reported an excellent antagonistic knack of paraprobiotics (heat-killed form) over the live probiotic bacteria against enteropathogens [38]. Moreover, the outcome of a recent literature survey by Pique and coworkers [39] underscores that postbiotics exert several pharmacodynamic features over live bacteria as enlisted below,

1. No risk of bacterial translocation from the gut lumen to blood among vulnerable and immunocompromised subjects.
2. No chances of acquisition and transfer of antibiotic resistance genes.
3. More natural to extract, standardize, transport, and store.
4. Loss of viability by cell lysis can produce further beneficial effects.
5. Enhanced interaction of every released molecule from the disrupted cells with the epithelial cells more directly.

## The forms of postbiotics and paraprobiotics

**Fig. 1:**
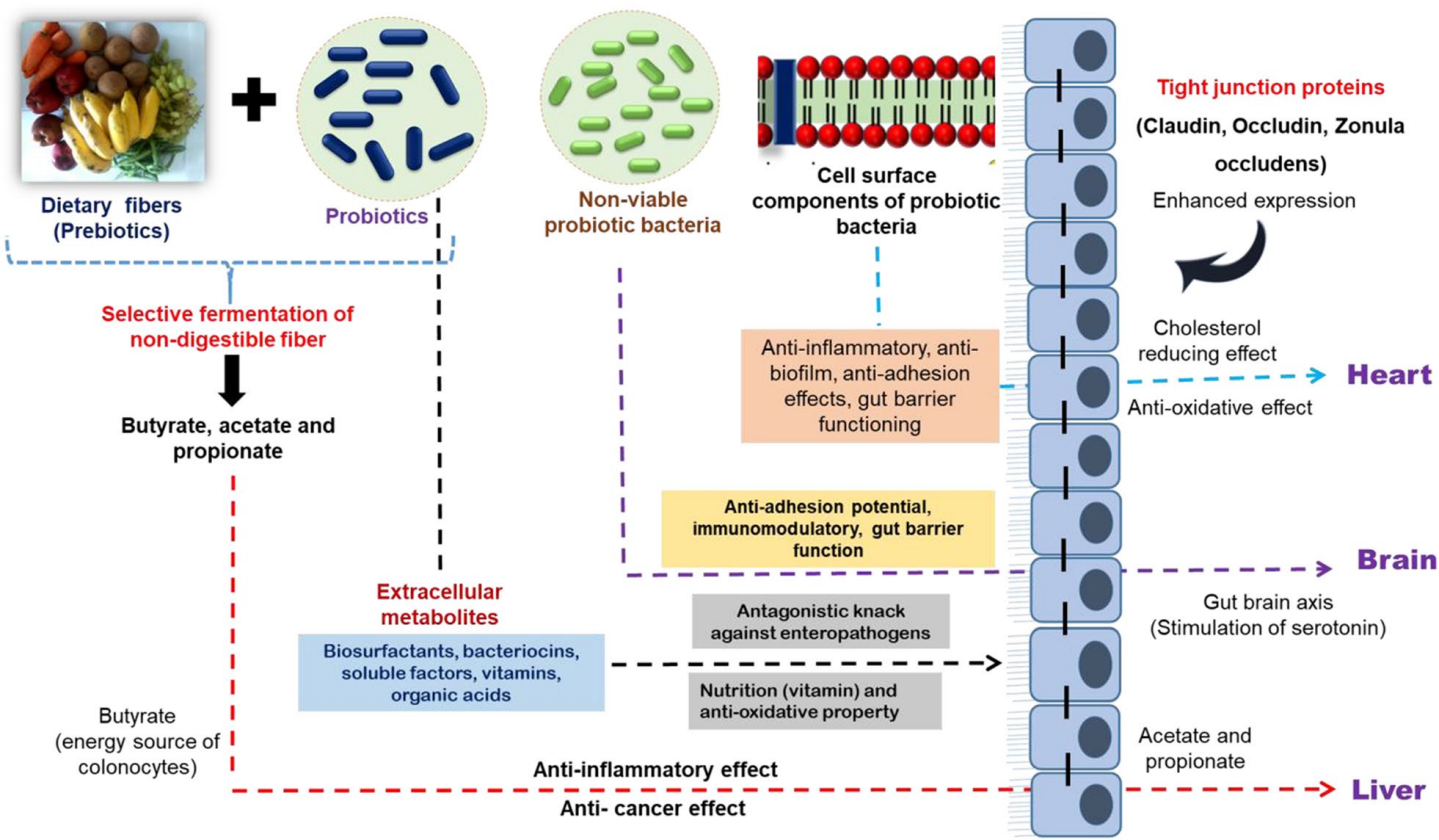
Schematic representation of various health benefits of postbiotic molecules

The various postbiotic molecules include metabolic by-products of live probiotic bacteria such as cell-free supernatant, vitamins, organic acids, short-chain fatty acids, secreted proteins/peptides, bacteriocins, neurotransmitters, secreted biosurfactants, amino acids, flavonoids derived postbiotics (desaminotyrosine, equol daidzein, daidzein, norathyriol), terpenoids derived postbiotics (genipin, paeoniflorin, paeoni lactone glycosides, paeonimetabolin I, II, III), phenolic-derived postbiotics (equol, urolithins, valerolactones, enterolactone, enterodiol, 8-prenylnaringenin) etc. [40–42]. Whereas, the paraprobiotics constitutes inactivated/dead/non-viable microbial cells of probiotics as intact or ruptured containing cell components of probiotic cells upon lysis such as teichoic acids, peptidoglycan-derived muropeptides, surface protruding molecules (pili, fimbriae, flagella), polysaccharides like exopolysaccharides, cell surface-associated proteins, cell wall-bound biosurfactants, teichoic acids, etc. [24, 43]. The use of purified postbiotic components or individual cellular components for therapeutic studies targeting a particular disease helps to rule out the specific underlying molecular mechanisms displayed by each molecule. However, to study the same in probiotics may results in unclear and multiple outcomes due to complex bacterial architecture/morphology. Therefore, various postbiotic molecules have drawn attention due to their known chemical structure, long storage stability, and the ability to trigger the various mechanisms in controlling inflammation, adhesion of pathogens to GIT, obesity, hypertension, coronary artery diseases (CVD), cancer, and oxidative stress (Fig. 1). Fascinatingly, postbiotic preparations have also granted patents as bio-therapeutics for a specific health claim “immune-modulation” [44, 45]. Similarly, metabolites of lactic acid bacteria (postbiotics) have granted patents as anti-tumour agents and feed additives for monogastric animals [46–48].

### Non-viable probiotics

**Fig. 2:**
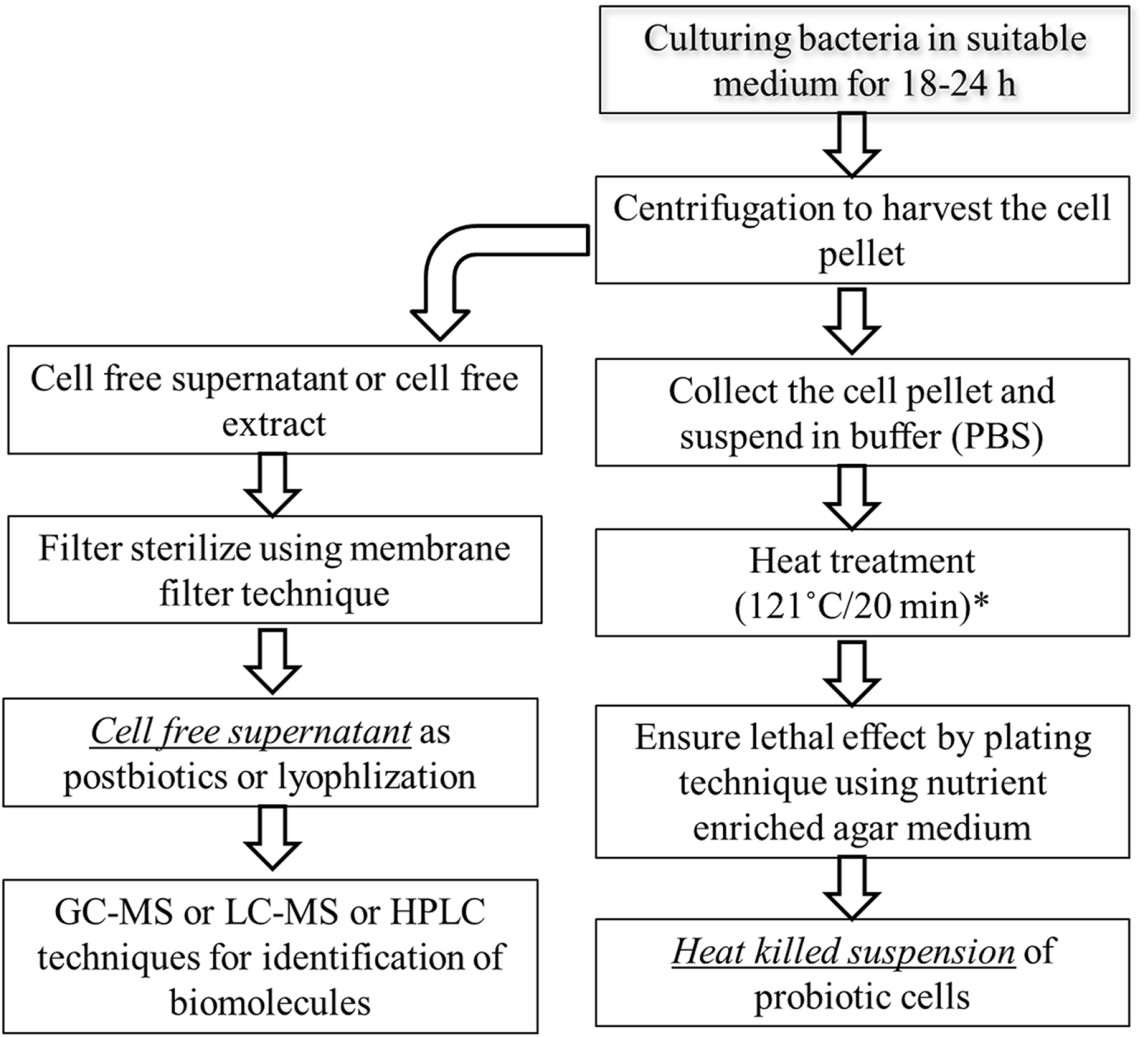
Process flow line for production and characterization of heat killed cells and cell free supernatant [170]. (* denote that the use of different time–temperature combinations to ensure complete lethal effect)

The non-viable probiotics are the inactivated or dead cells of probiotics. The inactivation of live bacteria can be achieved by various methods viz. heat treatment, chemicals (e.g., formalin), gamma or ultraviolet irradiation, and sonication, however, most commonly, heat treatment remains the method of choice for inactivation [32]. Nevertheless, the mode of inactivation by different methods, their effect on cellular structural components, and their influence on biological activities remain non-identical [49]. Heat treatments comprise a wide range of time-temperatures combinations to ensure the complete killing of bacteria in the suspension (Fig. 2). The inactivation can also be achieved by a combination of tyndallization and cell freezing process. In a study, the bacterial heat-killed suspension was prepared by heating cell suspension (10^9^ CFU/mL) to 100 °C/30 min, and the lethal effect was confirmed by pour plating [50]. Regarding the confirmation of heat-induced lethal effect and to understand the heat-induced changes on structural confirmations of various biomolecules, Attenuated Total Reflection- Fourier-transform Infrared Spectroscopy (ATR-FTIR) technique was successfully employed in a study by considering the specific peak at 1635 cm^−1^ (amides of proteins and peptides) [51].

It is important to note that non-viable cells maintained their potentiality to deliver beneficial effects on the host at the intestinal level in vivo, thus aid the development of safer preparations with more optimal pharmaceutical properties [37, 52]. Heat-killed probiotics revealed anti-adhesion (competition for adhesion sites) ability against various enteropathogens on the Caco-2 experimental model [38]. This promising potentiality of heat-killed probiotics may indicate the ability to fight against diarrheal and food-borne pathogens in terms of their ability to compete for adhesion sites on gut enterocytes. Additionally, several heat-killed probiotics strains of *Lactobacillus* have demonstrated the anti-inflammatory (ability to suppress the inflammatory markers like IL-6, TNF- α and to enhance anti-inflammatory cytokines viz. IL-10) and anti-oxidative (ability to scavenge the free radicals) effects at in vitro and in vivo experimental models [35, 53]. On the other hand, probiotic heat-killed preparation had a substantial influence over serotonin secretion in the gut (gut-brain axis) [54]. A study by Saito et al. demonstrated that oral administration of heat-killed preparations of *L. brevis* SBC8803 to rats upregulates the acyl ghrelin concentration that in turn increased the ratio of acyl to des-acyl (inactive) ghrelin in blood. Apart from this, heat-killed cells also enhanced the expression of the *Syt3* (synaptotagmin 3) gene related to ghrelin exocytosis in primary mouse stomach cells [55]. These findings suggest that not only live bacteria but also their heat-killed cells have the caliber to modulate host physiology. Moreover, there have been several lines of evidence currently available to demonstrate the similar mode of action of heat-killed probiotics vis-à-vis viable cells [36, 52, 56].

### Biosurfactants

**Fig. 3:**
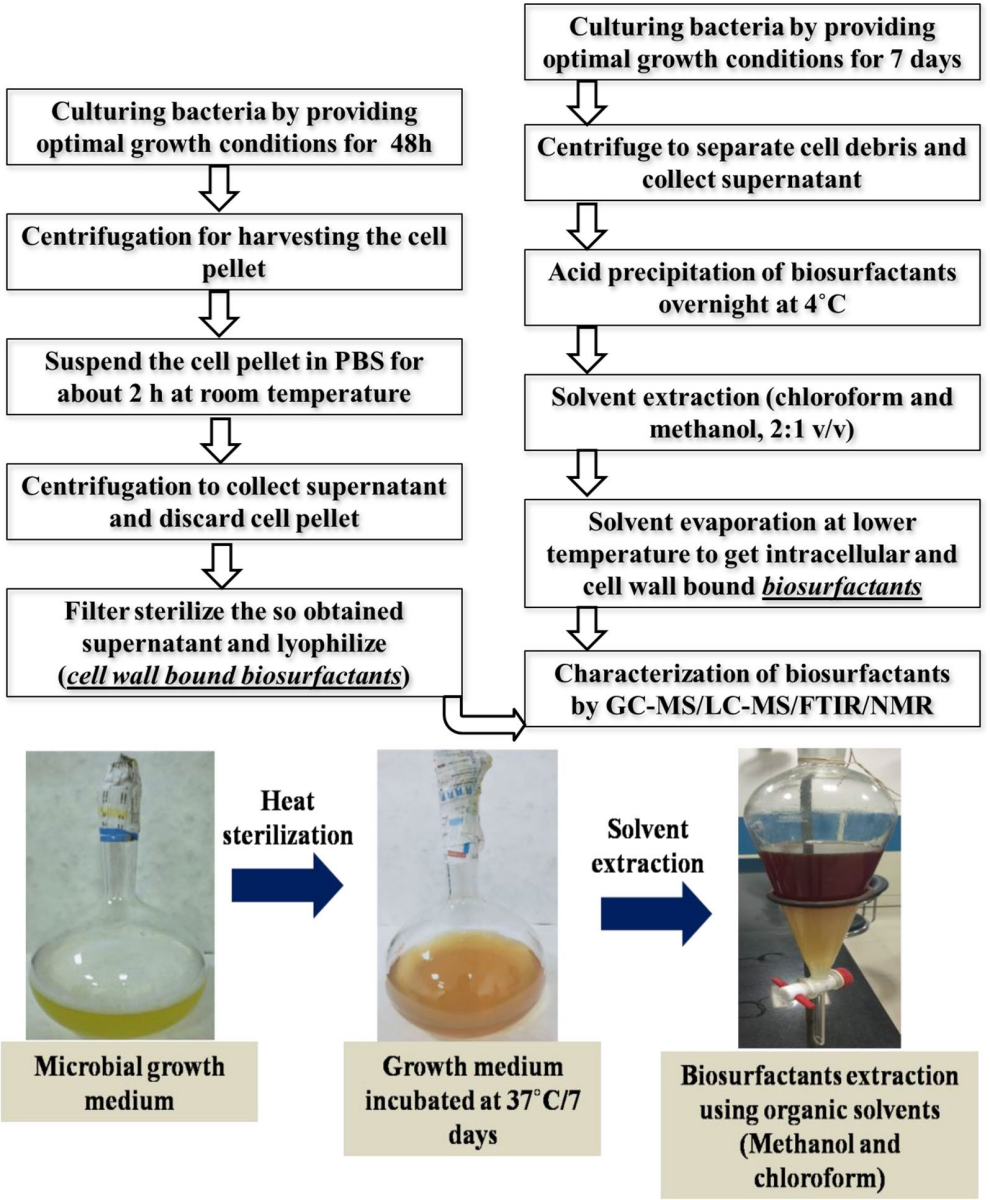
Production and characterization flow line of cell wall bound and intracellular biosurfactants [77, 171, 172]

Biosurfactants (BS) are the diverse polymeric molecules synthesized during the late log or early stationary phase of the growth cycle of an organism and are secreted extracellularly or cell wall-bound. BS assists the own cell in (i) nutrient uptake by increasing the surface area (ii) hydrophobic substrate metabolism (iii) cellular defense mechanism [57, 58]. Amongst the plethora of BS, few representatives viz. glycolipids, lipopeptides, phospholipids, neutral lipids, polysaccharide-protein complexes, and free fatty acids have been well documented [59, 60]. BS are the amphiphilic molecules comprising hydrophobic (fatty acids or hydrocarbon chain) and hydrophilic (polysaccharide or peptides or acids) moieties to provide surface-active and emulsification properties that reduce the interfacial tension at the surface. This amphiphilic property of BS assists in the disruption of pre-formed biofilms or preventing the onset of biofilm formation by pathogenic microorganisms. Also, the wetting, foaming, and emulsification properties hurdle the bacteria to adhere, establish, and subsequently to communicate in the biofilms [61]. Studies have defined the possible insights on antagonistic actions of BS agaisnt bacterial cells which include (i) the interaction of hydrophobic moieties of BS with membrane lipid to affect membrane integrity by pore formation (ii) direct interaction with the membrane lipids to trigger inhibition of the membrane-confined enzyme to cause outflow of intracellular cytoplasmic components [62, 63]. The BS extraction methodology involves the solvent extraction principle (Fig. 3). The use of chloroform and methanol (2:1, v/v) helps to recover both non-polar and polar molecules, respectively, from the bacterial fermentate. This method recovers both intracellular and cell-wall bound BS [64]. Furthermore, the characterization of extracted BS can be achieved by FTIR, Nuclear Magnetic Resonance (NMR), and other chromatographic techniques like Gas Chromatography-Mass Spectrometry (GC–MS), Liquid Chromatography-Mass Spectrometry (LC–MS). To minimize the cost of production, researchers have exploited agricultural by-products based medium (substrate) which has markedly hiked the productivity of biosurfactants. Hence, technology interventions to scale-up such methodologies would perhaps overcome the cost constraint for industrial partners to implement the production process [65].

BS offers several advantages over commercial detergents such as food grade, higher biodegradability, low toxicity, stability at various processing parameters like pH, temperature, and salt concentrations [66, 67]. However, few factors like feasibility, low yield, high cost of production are currently restricting production at commercial scale. Biotechnological intervention in strain improvement and optimization of agro-by-products based media may overcome the aforementioned drawbacks. Despite such limitations, rhamnolipids, sophorolipids, and mannosyl erythritol lipids (MELs) have been reported to produce at the commercial scale [68]. As far as food, pharmaceutical, and biomedical applications are concerned, the properties like emulsion stabilization, anti-adhesion, anti-biofilm, anti-cancer, anti-viral, immunomodulatory and antimicrobial abilities have been exploited [69] (Table 1).

**Table 1:** Different health benefits of paraprobiotic and postbiotic molecules

Probiotic organisms	Paraprobiotic/postbiotic components	Type/model of study	Health benefits	Method of isolation and characterization	Refs.
*L. rhamnosus* GG	Heat killed cells	Rat model	Anti-inflammatory	Heat treatment (80 °C for 20 min)	[174]
*L. brevis* SBC8803, *L. brevis* -8013 *B. longum,* and *Streptococcus faecalis*	Heat killed cells	Caco-2 cells and colitis mouse model	Anti-inflammatory and Enhancement of epithelial barrier permeability	Heat treatment (121 °C for 20 min)	[170]
*L. paracasei* spp. *paracasei* (strain 06TCa22) and *L. plantarum* (strain 06CC2)	Heat killed cells	*In vitro* kidney cell line	Immunomodulation	Heat treatment (100 °C for 1 h)	[175]
VSL#3 (*B. breve*, *B. longum,* and *B. infantis*; *L. plantarum*, *L. bulgaricus*, *L. casei* and *L. acidophilus*; *S. salivarius* subsp. *thermophilus*)	Heat killed cells	Dextran Sodium Sulfate (DSS) induced colitis rat model	Anti-inflammatory	Heat treatment (121 °C for 20 min)	[176]
*Lactobacillus* spp.	Heat killed cells	*In vitro*	Anti-biofilm effect against oral pathogens	Heat treatment (100 °C for 3 min)	[177]
*L. casei* and *L. rhamnosus* GG	Cell-free supernatant	HCT-116 cell line	Anti-cancer effect, Anti-inflammatory, Enhancement of gut barrier property	–	[178]
*Lactobacillus* spp.	Cell-free supernatant	Vaginal epithelial cells	Anti-adhesion effect against *E. coli*	–	[179]
*L. paraplantarum*	Cell-free supernatant	*In vitro*	Antagonistic effects against MRSA	HPLC (organic acids identification)	[180]
*L. rhamnosus* GR-1	Cell-free supernatant	Amnion cells	Immunomodulatory activity	–	[181]
*L. rhamnosus* GG	Cell-free supernatant	Caco-2 cell line	Protective effect against the *E. coli* induced barrier dysfunctioning by influencing the expression of MUC2, ZO-1, IgA, mucin	–	[182]
*Lactobacillus* isolates	Cell-free supernatant (antimicrobial proteins)	In vitro	Suppression of multidrug-resistant *Helicobacter pylori*	Purification and identification by reverse phase-HPLC	[183]
*Weissella cibaria* strain CMU (oraCMU),	Cell-free supernatant	In vitro	Antagonistic effects against *Porphyromonas gingivalis* (an oral pathogen)	Identification of lactic acid, acetic acid, and citric acid by HPLC; Short-chain fatty acids by GC–MS; Secretory protein by 2D-gel electrophoresis and MALDI-TOF/MS	[184]
*L. fermentum*	Cell-free supernatant	3T3-L1 pre-adipocytes	Anti-senescence potential (alleviated senescence markers viz. p53, p21^WAF1^, SA-β-gal, p38MAPK, iNOS, cox-2, ROS, NF-κB, and DNA damage response)	–	[185]
*L. delbrueckii* subsp. *bulgaricus*	EPS	In vitro	Cholesterol-lowering effect	–	[186]
*Pediococcus parvulus*	EPS	LDL-receptor deficient mice model	Cholesterol-lowering and immunomodulatory effect	Ethanol precipitation method	[187]
*B. breve* BASO-1 and *Lactobacillus* spp.	EPS	*In vitro*	Anti-biofilm effect against *B. cereus*, *L. monocytogenes*, *E. fecalis*, *S. aureus*, *E. coli* 0157:H7, *E. coli* ATCC 35218, *P. aeruginosa*, and *Salmonella* spp	TCA and ethanol precipitation method	[188]
*L.* *fermentum* S1	EPS	In vitro	Anti-oxidative and anti-biofilm effect against *E. coli* and *S. aureus*	TCA and ethanol precipitation method. Further purification by anion-exchange chromatography	[189]
*L. jensenii* P6A and *L. gasseri* P6	Biosurfactants	In vitro	Anti-biofilm and anti-microbial effects against *E. coli, Candidaalbicans*, *Staph*. *saprophyticus*, *Enterobacter aerogenes*, and *Klebsiella pneumonia*	Cell wall-bound biosurfactants extraction in phosphate-buffered saline (PBS)	[66]
*L. casei* B1	Biosurfactants	In vitro human epithelial cell line (HEp-2)	Anti-oxidative, anti-proliferative, and anti-adhesion activity against *S. aureus*	Cell wall-bound biosurfactants extraction in phosphate-buffered saline	[190]
*Lactobacillus spp.*	Biosurfactants	In vitro	Antagonistic effects against *E. coli*	Chloroform and methanol extraction	[191]
*L. gasseri*	Biosurfactants	In vitro	Antibiofilm ability against methicillin-resistant *S. aureus* (MRSA)	Cell wall-bound biosurfactants extraction in phosphate-buffered saline	[192]
*L. paracasei* D3-5 strain	LTA	In vivo	Anti-aging	Cell disruption by mechanical method and LTA extraction by butanol method	[103]
*L. plantarum*	LTA	In vitro	Antibiofilm activity against *S*. *mutans*	Mechanical cell disruption method of extraction followed by MALDI-TOF/MS based structure confirmation	[108]
*L. plantarum*	LTA	In vitro	Anti-adhesion and antimicrobial effects	Mechanical disruption of cells, n-butanol extraction followed by confirmation of LTA by conventional silver staining after polyacrylamide gel electrophoresis	[193]
*L. plantarum* K8	LTA	HaCaT Cells and Mice model	Inhibition of TNF-**α** and IFNγ mediated C3 mRNA expression	-do-	[194]
*L. casei*	Peptidoglycan	Various cancer cell lines and CD-1 Swiss mice	Anti-tumour effect	Treatment of cell pellet with penicillin and d-glucose and subsequent collection of soluble peptidoglycan after centrifugation	[195]
*L. rhamnosus* CRL1505	Peptidoglycan	Immunocompromised-malnourished mice with *Streptococcus pneumoniae* infection	Enhancement of Th2 response	Mechanical disruption, delipidated by solvent extraction, and nuclease treatment	[196]
*L. kefir*	Surface layer proteins	Vero cells	Melioration of *Clostridium difficile* induced cytotoxicity	5 M LiCl extraction method, and confirmation by SDA-PAGE	[173]
*L. plantarum* 423	Surface proteins	Caco-2 cells	Anti-adhesion effect against *Clostridium sporogenes* and *Enterococcus faecalis*	4 M guanidine HCl treatment followed by 5 M LiCl based extraction, and SDS-PAGE confirmation	[197]
*L. acidophilus* CICC6074	S-layer proteins	Caco-2 cells and colitis mouse model	Lowered the intestinal epithelial apoptosis, with decreasing the IL-6 secretion	LiCl extraction and purification by Sephadex G-75 gel filtration column. Additionally, SDS-PAGE based molecular weight confirmation	[198]
*L. plantarum*	Mucus binding protein	*In vitro* Caco-2 and HT-29 cell line	Mucoantiadhesion and anti-colonization effects against *E. coli*	Cloning and expression of Mubs5s6 gene in *E. coli* followed by purification and confirmation by chromatography and SDS-PAGE based techniques	[94]
*L. acidophilus* DDS-1	SCFA	Aging C57BL/6 J mice	Increases in short-chain fatty acids (butyrate, propionate, and acetate) levels	GC–MS	[199]
*Lactobacillus spp*	SCFA	Mice	Increases in butyrate and propionate levels with ameliorate gut microbiome dysbiosis	HPLC	[200]
*L. rhamnosus* strain ASCC 1520	SCFA	BALB/c mice	Gut microbiota alteration	QTRAP LC–MS/MS	[201]
*B*. *bifidum*	SCFA (acetate)	Caco-2	Increase in TEER values	NMR	[202]

### Exopolysaccharides

Exopolysaccharides (EPS) are extracellular biopolymer synthesized or secreted by microorganisms during their growth; they widely vary in their degree of branching from linear molecules to highly branched molecules, and in monosaccharide composition [70]. Based on the monosaccharide composition, EPS is further classified into homo-polysaccharide having identical monosaccharide units (e.g. cellulose and dextran) and hetero-polysaccharide with different monosaccharides (e.g. xanthan) [71]. Hitherto, ample lactic cultures (*Lactobacillus fermentum, L. rhamnosus, Streptococcus thermophilus, Pediococcus pentosaceus, L. delbrueckii* subsp. *bulgaricus*, *Leuconostoc* species, etc.) have reported synthesizing EPS [72–74]. Nevertheless, the production of EPS is highly strain-specific behavior and depends on various factors like the composition of the medium, age of the cell, pH, and temperature [75]. The EPS of selected strains of dairy starter cultures is indeed a boon to the dairy industry as EPS has a strong command over the rheological properties of fermented dairy products since EPS gets hydrated and reduces moisture content [76]. The desired rheology of the products can be achieved either by the EPS production by starter cultures in situ or via the external addition of extracted and purified EPS.

**Fig. 4:**
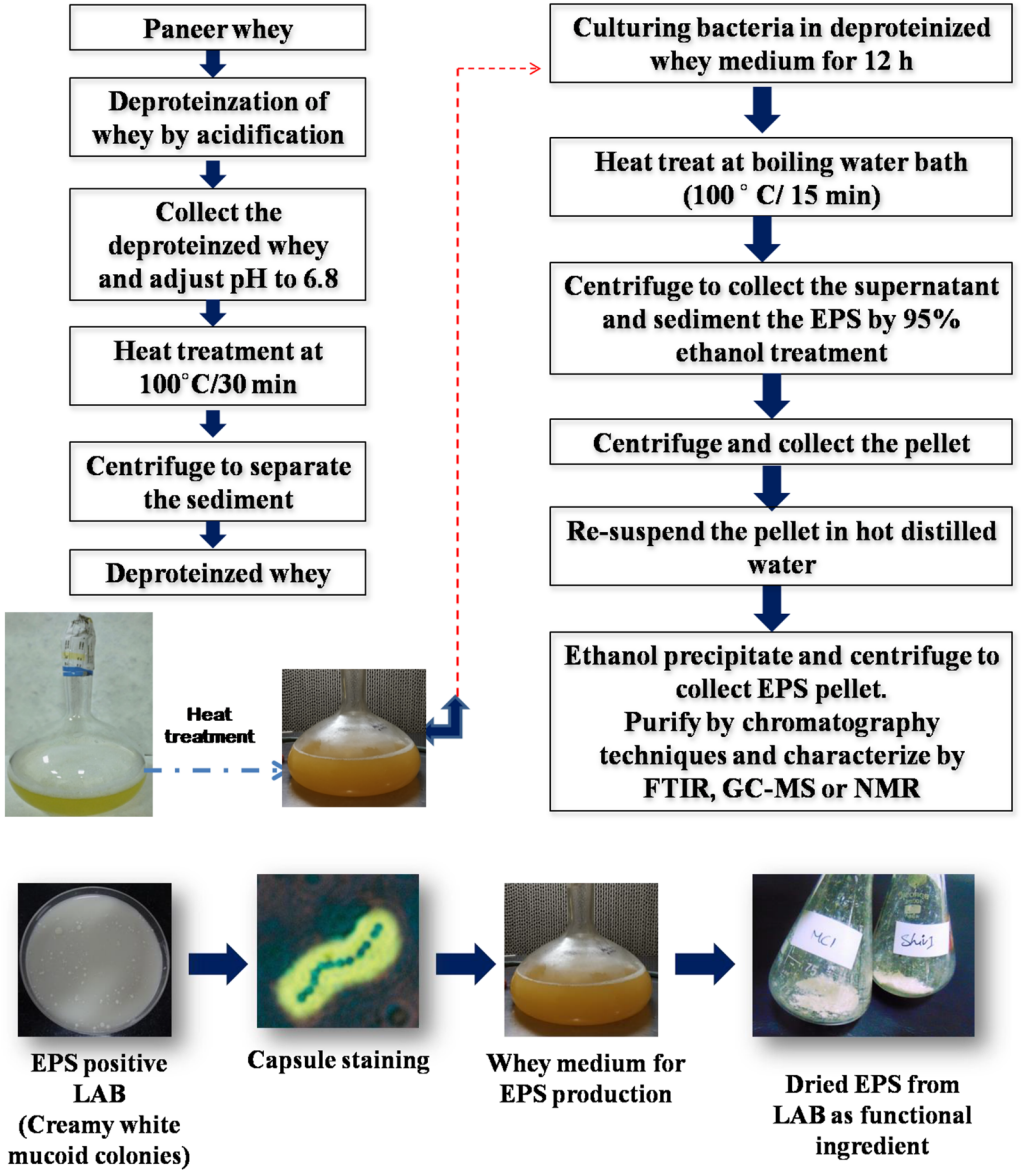
An outline on extraction and characterization of EPS from whey-based medium [74]

The extraction protocol of EPS includes culturing the EPS producing lactic acid bacteria (LAB) (De Man, Rogosa, and Sharpe/MRS for lactobacilli) in a suitable medium for 12 to 18 h and subsequent deproteinization of culture supernatant by trichloroacetic acid (TCA) and ethanol precipitation [77]. Since industrial production of EPS cannot relay on the MRS medium, investigators have optimized dairy whey for the sustainable production of EPS from lactic cultures (Fig. 4). On the other hand, purification of extracted EPS can be achieved by chromatography methods, and functional group characterization can be achieved by GC–MS, FTIR spectroscopy, or NMR techniques [78]. The EPS of lactic acid bacteria has also reported exhibiting several biofunctional attributes like anti-oxidative (ability to scavenge wide range of free radicals), cholesterol-lowering (ability to bind free cholesterol) effect, immunomodulatory effect, anti-aging effect, gut microbiota modulation, anti-toxic effect, anti-biofilm effect, and antitumor effects at preclinical trials [78–83].

### Cell surface proteins

**Fig. 5:**
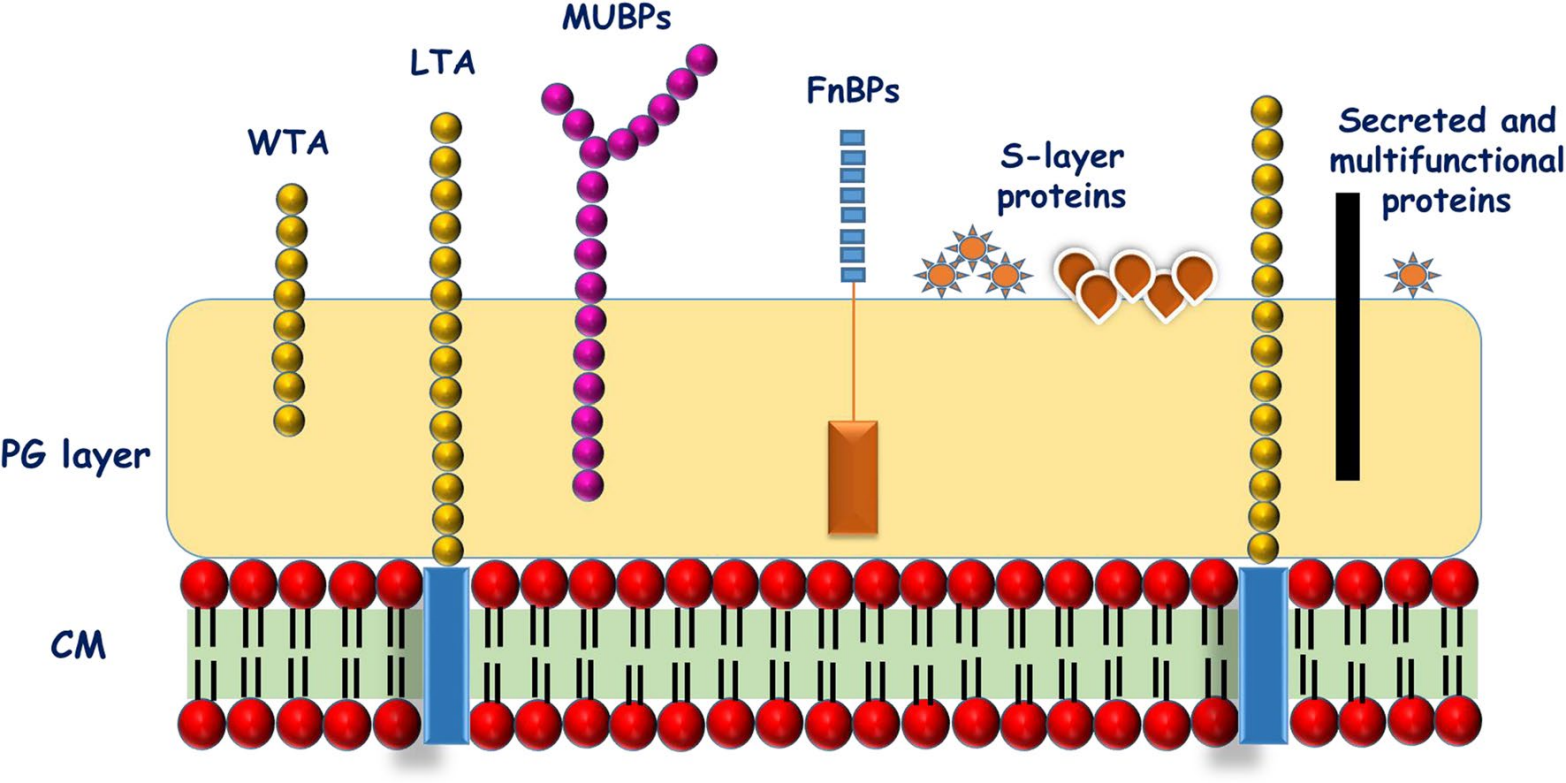
Diagrammatic representation of various cell surface-associated components of lactic acid bacteria. (CM, Cell membrane; PG, Peptidoglycan; WTA, Wall teichoic acids; LTA, Lipoteichoic acid; MUBPs, Mucin binding proteins), FnBPs, Fibronectin binding proteins; S-layer, Surface layer)(CM, Cell membrane; PG, Peptidoglycan; WTA, Wall teichoic acids; LTA, Lipoteichoic acid; MUBPs, Mucin binding proteins), FnBPs, Fibronectin binding proteins; S-layer, Surface layer)

The cell surface proteome of bacterial architecture plays a crucial role in exhibiting the dynamic molecular mechanism of probiotics. The bacterial surface proteins are classified in four categories, which include (i) proteins anchored to the cytoplasmic membrane by hydrophobic transmembrane domains (integral membrane proteins), (ii) lipoproteins (covalently attached to membrane lipids after cleavage of a signal peptide by signal peptidase II), (iii) proteins containing C-terminal LPXTG-like motif and covalently attached to peptidoglycan by sortases, and (iv) non-covalently bound proteins associated with cell wall by weak interactions (van der Waals forces, hydrogen or ion bonds) (LysM proteins, WXL proteins, GW proteins, proteins with choline-binding domains) [84]. The well-characterized cell surface-associated proteins in probiotic bacteria include surface (S) layer protein, mucus binding protein, fibronectin-binding protein, sortase dependent binding protein, collagen-binding protein, and so on (Fig. 5). These surface proteins are vital in probiotic bacterial adhesion to host econiche. Indeed, the probiotic lactobacilli lacking S-layer proteins witnessed the inferior adhesion knack to the gut enterocyte [85]. The cell surface-associated proteins constitute the first-line of contact during the potential interplay with the host which in turn trigger the various cellular process (signal transduction mechanisms) in the intestinal cells involving nuclear factor-κB (NFκB) and mitogen-activated protein kinases (MAPKs). This influences the regulation of downstream pathways such as the secretion of cytokines (chemokines and cytokines) which are responsible for the immunomodulatory action, secretion of antibacterial peptides (defensins), mucin secretion, expression of tight junctions factors, etc. [86–88].

**Fig. 6:**
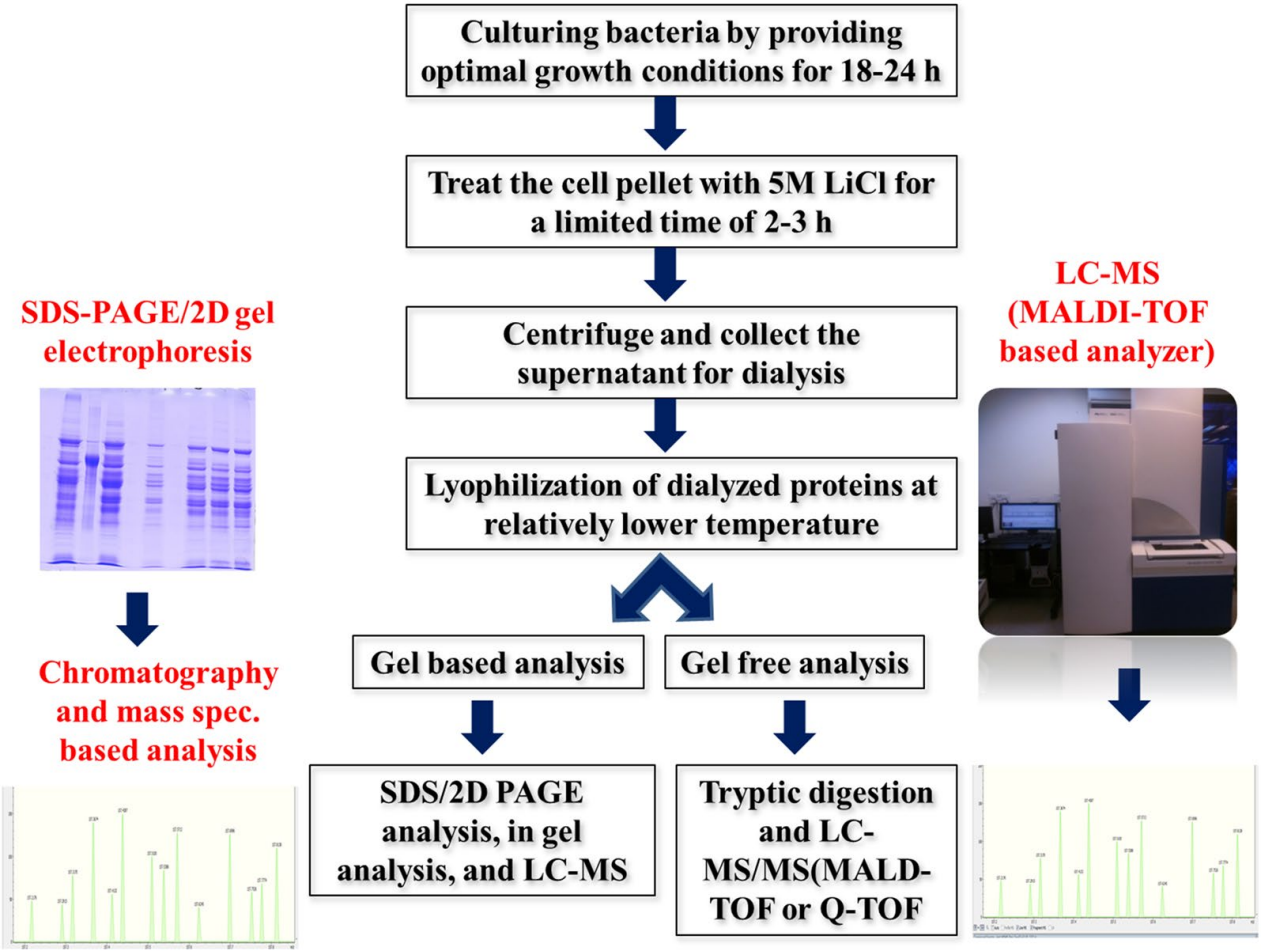
Brief overview of the extraction and characterization of surface proteins associated with lactobacilli [173]

The enzymes (EDTA-lysozyme) and chemical chaotropic agents [lithium chloride, guanidinium hydrochloride, urea, and sodium dodecyl sulphate (SDS)] have been widely reported to shave the surface proteins [84]. The gel-based and non-gel based methods have been widely employed to characterize the extractable proteins (Fig. 6). The gel-based proteomics techniques include separation of surface proteins on Sodium Dodecyl Sulphate–Polyacrylamide Gel Electrophoresis (SDS-PAGE) or two-dimensional (2D) gel electrophoresis and identification of bands by Liquid Chromatography-Mass Spectrometry (LC–MS) (Matrix-Assisted Laser Desorption/Ionization-Time of flight, MALDI-TOF; or Quad Time of Flight, ESI-Q-TOF) upon tryptic digestion (in gel analysis). However, the gel-free proteome identification constituted the direct digestion of surface proteins with trypsin and analysis of tryptic peptide by LC–MS/MS (short gun proteomics) techniques and followed by bio-informatics analysis [89].

So far, the surface proteins of probiotic bacteria have reported demonstrating anti-inflammatory, anti-adhesion, strengthening the epithelial barrier property, and biosorption of toxic heavy metals [90]. The purified surface proteins of *L. helveticus* significantly hampered the adhesion of *Escherichia coli* O157:H7 and enhanced the transepithelial epithelial electrical resistance (TEER) which was initially decreased by *E. coli* infection in Hep-2 and T84 human epithelial cell lines [91]. In contrast, the extractable surface proteins of *L. plantarum* ameliorated the pathogen invasion by signaling the expression of tight junction proteins viz. Claudin-1, Occludin, JAM-1, and ZO-1, and thus restored the epithelium integrity [88, 92]. More recently, the extractable surface proteins of (S-layer proteins) of probiotic *Enterococcus faecium* WEFA23 significantly declined the *Listeria monocytogenes* induced apoptosis of Caco-2 [93]. Mucus binding proteins (MUB) of *L*. *plantarum* hampered the adhesion of *E. coli* to the gastric mucin and HT-29 cell line [94]. Apart from pathogen exclusion, the immunomodulation potential of surface proteins is also well documented [95]. The surface proteins of *L. rhanmosus* GG revealed to alleviate the inflammatory cytokines and TLR activation at mRNA level in the in vitro cell line model. Moreover, the surface proteins have attenuated lipopolysaccharides (LPS)-induced MAPK and NF-kB signaling pathways activations in the intestinal epithelial cell (IEC) IPEC-J2 [96]. These findings suggest that surface proteins of probiotic bacteria may be the better biotherapeutics in the inflammatory diseases like inflammatory bowel disease (IBD) or colitis.

### Teichoic acids

The surface charge on the peptidoglycan layer of gram-positive bacteria is pivoted towards the presence of anionic glycopolymers called wall teichoic acids (WTAs). These play key roles in determining the cell shape, regulation of cell division, and other fundamental metabolic aspects of cell physiology. In addition, teichoic acids also confer pathogenesis and antibiotic resistance to gram-positive bacteria [97–99]. The teichoic acids are chemically the glycopolymers (ribitol) containing phosphodiester-linked polyol repeat units. Teichoic acids are generally of two kinds, which include lipoteichoic acids (LTAs) (anchored in the bacterial membrane via a glycolipid), and wall teichoic acids (WTAs) (covalently attached to peptidoglycan) [100]. The wall teichoic acid polymer can be divided into two components, a disaccharide linkage unit and the main chain polymer composed of phosphodiester-linked polyol repeat units. LTA is an amphiphilic molecule in which the hydrophilic region is made of 1,3-phosphodiester-linked polymer of glycerol-phosphate or ribitol-phosphate substituted with d-alanine or sugars. In contrast, the hydrophobic region is a glycolipid (Glc (β1-6) Glc (β1-3) diacylglycerol) [101].

The extraction protocol of LTA involves the mechanical disruption of the cell wall of bacteria by sonication in the citrate buffer (0.1 M, pH = 4) to release the teichoic acids from the peptidoglycan layer. The lipophilic cell contaminants from the LTA can be overcome by solvent (n-butanol) extraction. The obtained crude LTA can be purified by gradient hydrophobic chromatography using octylSepharose CL-4B packed in XK 16/60 column with 15% to 60% n-propanol in ammonium acetate (0.1 M, pH = 4.75) as mobile phase. To achieve further purity, the collected fractions can be further analyzed for their phosphate content using the phosphomolybdenum test. Nevertheless, the Nuclear Magnetic Resonance (NMR) technique was also employed to study the structural insights of LTA [101].

Ample studies have been conducted to study the functional attributes of LTA, out of which, immunomodulatory potential has much underpinned. LTA is a microbe-associated molecular pattern (MAMP) recognized by pattern recognition receptor (TLR-2) on the surface of gut enterocytes to transduce cellular signals to induce inflammatory cytokines response [102]. On the contrary, the administration of LTA extracted from *L. paracasei* D3-5 strain to old mice ameliorated the high- fat diet-induced metabolic dysfunctions, decreased leaky gut and inflammation, and improved physical and cognitive functions. Moreover, LTA also stimulated the expression of mucin (*Muc2*) gene by modulating TLR-2/p38-MAPK/NF-kB pathway [103]. The LTA from *L. delbrueckii*, *L. sakei*, and *L. rhamnosus* GG suppressed the viral double-stranded RNA (poly I:C) induced pro-inflammatory cytokine (IL-8) in the intestinal epithelial cell line. In contrast, the dealanylated or deacylated LTA did not show the signs of poly I:C-induced IL-8 production, this difference may counsel the role of d-alanine and lipid moieties in contributing functional attributes to LTA structure [104]. In another study, the LTA from the *L. rhamnosus* GG showed dose-dependent activation of NF-κB signaling in HEK293T (intestinal cell line) and Caco-2 cells after interaction with TLR2/6, but not with TLR2 alone. Besides, the experiments with highly purified LTA of LGG resulted in the IL-8 (pro-inflammatory cytokine) mRNA induction in Caco-2 epithelial cells, whereas the process of dealanylation and deacylation of LTA reduced IL-8 mRNA expression [101]. A study by Ahn et al. demonstrated that LTA extracted from *L. plantarum* K8 was found to regulate the balance between Th1 and Th2 response (pro and anti-inflammatory response cytokines). The homeostasis was noticed between IL-10 and TNF- α upon treatment to phorbol-12-myristate-13-acetate (PMA)-differentiated THP-1 cells (macrophages) with the LTA. Interestingly, this response was found to be highly strain-specific, and the authors failed to observe the same outcomes with LTA of *Staphylococcus aureus* or *L. sake*, hence, therapeutic capabilities of LTA can be inferred only after proper examination in suitable models to use LTA [105]. Nevertheless, the cytokine-induced immunomodulatory activity of LTA is further debatable and required a large number of well-designed experimental models to rule out the specific outcome.

On the other hand, LTA of lactobacilli has been also studied as biofilm disrupting agents. In this connection, the purified LTA extracted from probiotic *Lactobacillus* strains have demonstrated anti-biofilm actions against oral and enteric pathogens such as *Streptococcus mutans, S. aureus*, and *E. feacalis* by preventing the formation of biofilms and disrupting the preformed biofilms [106–108]. Likewise, LTA of *L. plantarum* could curb the biofilm formation and aggregation without affecting the growth of *S. aureus* in various in vitro and in vivo models [109]. However, it is important to note that the same effect was not observed upon the removal of d-alanine moieties from the LTA structure. Therefore, it is crystal clear that the d-alanine structure in LTA is crucial to provide various functional properties. The in-depth investigation on the mechanism of antibiofilm action of LTA against *S. aureus* demonstrated the control of LTA over *ica*-operon which is responsible for the production of poly-*N*-acetylglucosamine (the key molecule in *S. aureus* biofilm development). Moreover, LTA increased the release of autoinducer-2 from *S. aureus*, which contributes to the inhibition of *S. aureu* biofilm formation. On the other side, LTA treatment enhanced the susceptibility of the biofilm to various antibiotics and macrophages [109].

### Peptidoglycan

The peptidoglycan (PG) sacculus is an indispensable cell wall structural element present most abundantly among Gram-positive bacteria. The peptidoglycan is a linear glycan strand cross-linked by peptides. The peptidoglycan strands are constructed by bonding N-acetylglucosamine (GlcNAc) and N-acetylmuramic acid (MurNAc) residues via beta 1-4 linkages. The peptide chains are linked covalently through their N-terminus to the lactyl group of MurNAc [110]. In contrast, the peptidoglycan structure in LAB is quite peculiar; the amino acid sequence of the stem peptide is L-Ala-g-D-Glu-y-D-Ala, while the third amino acid (y) is a di-amino acid. In some cases, it is often L-Lys (e.g., in *Lactococcus lactis* and most lactobacilli) but can also be mesodiaminopimelic acid (mDAP) (e.g., in *L. plantarum*) or l-ornithine (e.g., in *L. fermentum*). D-Lac, however, replaces D-Ala present at the position fifth position in the newly synthesized PG in a few LAB such as *L. casei*, *L. plantarum,* and *Leuconostoc* spp. that provide innate resistance to vancomycin [111]. Peptidoglycan extraction from probiotic lactobacilli is a multistep process that includes both mechanical and enzymatic separation. The obtained cell wall has to be delipidated by the successive solvent extraction method (methanol–chloroform (1:1)). Consequently, delipidated crude preparations should be enzymatically purified by treating with a cocktail of proteases and nucleases [112, 113]. Further, amino acids can be confirmed by SDS-PAGE and carbohydrates can be profiled by chromatographic or NMR techniques. The peptidoglycan extracted from probiotic bacteria demonstrated in vitro and in vivo anti-cancer effect [114, 115], in vivo immunomodulatory activity (peptidoglycan from *L. rhamnosus* CRL1505 significantly improved lung CD3^+^CD4^+^IFN-γ^+^, and CD3^+^CD4^+^IL-10^+^ T cells as well as CD11c^+^SiglecF^+^IFN-β^+^ alveolar macrophages with the consequent increases of IFN-γ, IL-10, and IFN-β in the respiratory tract) [112, 116], in vivo anti-inflammatory effect in a colitis mouse model [117]. To date, only the immunomodulatory and anti-proliferative or anti-tumour effects of probiotics mediated peptidoglycan have explored yet.

### Cell-free supernatant and soluble factors

A unique compositional profile of lactic acid bacteria (LAB) derived cell-free supernatant (CFS) is driving the critical interest among researchers looking at various biomolecules targeting to seek health-promoting properties. CFS of LAB is a consortium of low molecular weight (i.e. hydrogen peroxide, reuterin, organic acids, carbon dioxide, and di-acetylene) and high molecular weight (i.e. bacteriocins and bacteriocins-like substances) compounds which are generally known as metabolites [118–120]. However, the composition of postbiotic metabolites was found to be affected by individual nutrients in the growth medium [121]. The separation of CFS is a lucid technique (Fig. 2) that involves centrifugation (10,000×*g* for 10 min @ 4 °C) and membrane filtration (0.22 μm polyethersulfone membrane) of 24 h grown culture medium [122, 123]. However, the lyophilization or freeze-drying of sterile CFS remains the optional step [124].

The characterization of CFS of *L. salivarius*, *L. casei* 431, and *L. acidophilus* LA5 resulted in various metabolic byproducts like short-chain fatty acids, organic acids, hydrocarbons, phenol, amino acids, benzoic acids, alcohol, sugars, peptides, etc. [124]. The CFS of the aforementioned probiotic strains revealed anti-microbial and anti-biofilm caliber against *Listeria monocytogenus*, a zero-tolerance pathogen [124]. On the other hand, the other metabolites like phenyllactic acid and lactic acid extracted from *L. plantarum* CECT-221 revealed inhibitory activity against *Carnobacterium piscicola*, *S. aureus*, *Pseudomonas aeruginosa*, *Listeria monocytogenes,* and *Salmonella enterica*. Moreover, the volatiles profiling of CFS of the same probiotic strain revealed natural aromas, such as acetophenone, with a high price in the market [125]. CFS of *L. rhamnosus* GG modulated the mucin expression and anti‐inflammatory cytokines such as interleukin (IL)‐4, IL‐5, and IL‐10 in HT-29 cell line [126]. Additionally, the CFS of *L. acidophilus*, *L. casei*, *L. lactis*, *L. reuteri*, and *Saccharomyces boulardii* appeared to unveil the signs of anti-oxidative knack [127]. More interestingly, the cell-free soluble factors of *E. coli* Nissle 1917 demonstrated the protective effect against enteropathogenic *E. coli* induced intestinal epithelial barrier dysfunctioning by triggering the expression of tight junction (ZO-1, claudin-14, and claudin-2) gene expression in Caco-2 cellular model [128]. The CFS of the three strains (*L. rhamnosus* strains SHA111, SHA112, and SHA113 isolated from human breast milk) showed excellent antioxidant activity against DPPH free radicals, superoxide anion radicals, and hydroxyl radicals) and anticancer activity on cervix cancer cells (HeLa) via cytotoxicity and induction of apoptosis through up-regulation of BAD, BAX, Caspase 3, Caspase 8, Caspase 9, and down-regulation of *BCL*-*2* genes in HeLa cells [129]. In another study, a soluble protein of 12 kDa in CFS of *L. acidophilus* ATCC 43121 exhibited cholesterol-binding activity and thus indicating the cholesterol-lowering activity of postbiotics [130]. On the other hand, CFS of *L. acidophilus, L. casei*, *L. reuteri*, and *S. boulardii* were able to downregulate the expression of PGE-2 and IL-8 in human colon epithelial HT-29 cells. Moreover, probiotic supernatant differently modulate IL-1*β*, IL-6, TNF-*α*, and IL-10 production by human macrophages, suggesting a typical anti-inflammatory activity [131].

The biofilms of pathogenic bacteria are one of the significant threats to the medical fraternity. The encased bacteria in the biofilm matrix are resistant to different antimicrobials, and thus biofilm seems to be the foremost aspect of pathogenesis and therapeutic failure. In this aspect, several investigators have focused on exploring CFS as an anti-biofilm agent due to its amphiphilic chemical profile. The CFS extracted from *Lactobacillus* spp. could able to prohibit the onset of biofilm formation and also able to disrupt the preformed biofilms of *Cronobacter sakazakii* and *L. monocytogenes* [132]. Similarly, the pH neutralized CFS of *L. plantarum*, *L. helveticus*, *Propionibacterium acidilactici,* and *E. faecium* revealed a substantial reduction in biofilm formation of *S. aureus* CMCC26003 and *E. coli* CVCC230 [133]. To overcome biofilm-forming multi-drug resistant superbugs (*P. aeruginosa, S. aureus*, and *E. coli*), investigators have used CFS of LAB for the successful mitigation of pathological conditions [134, 135]. Therefore, these findings suggested that the CFS of LAB may act as bio-liquid-detergent that reduces the adhesion and biofilm formation of pathogens to the various surfaces (biotic and abiotic surfaces).

### Bacteriocins

Lactic acid bacteria (LAB) produce an array of extracellular antimicrobials that inhibit both pathogenic and spoilage causing microorganisms. In situ production of antimicrobials by protective lactic cultures made their food exploitations more compatible [136]. Although a wide range of microorganisms produces bacteriocins, those produced by LAB have attracted to a greater extent due to their extensive applications in food processing and food fermentations as natural bio-preservatives. For instance, they have been extensively used in the preservation of ample food products like cheese, paneer, meat, and vegetables [137]. Since the bacteriocins are secreted extracellularly, the CFS is used for isolation and purification of proteinous bacteriocins. Briefly, the proteinaceous CFS is precipitated with ammonium sulphate (60–80%), antagonism can be assayed by native PAGE and peptide sequence is identified by LC–MS/MS [138].

Bacteriocins are ribosomally synthesized antimicrobial peptides produced by both Gram-positive bacteria and Gram-negative bacteria that inhibit closely related species. Few authors classify bacteriocins into two broad categories viz; Class I bacteriocins that are RiPPs (Ribosomally Produced and Post-translationally modified Peptides) having unusual amino acids like lanthionine and β-methyl lanthionine; Class II that do not contain unusual modifications [139]. On the contrary, there have been several reports classifies the five different classes of bacteriocins. Class I: RiPPs (lantibiotics) having unusual amino acids, Class II: Unmodified bacteriocins (small heat-stable with less than 10 kDa), Class III: These are unmodified and larger than 30 kDa, Class IV: complex bacteriocins having lipid or carbohydrate moieties, Class V: circular bacteriocins [140, 141]. The bacteriocins produced by LAB are generally cationic peptides, which act on cytoplasmic membranes by forming pores [142]. This triggers the leakage of intracellular vital components. However, mechanism of action of lantibiotic (Class A) nisin and pediocin-like bacteriocins is on lipid-bound cell wall precursor lipid II as a docking molecule for subsequent inhibition of peptidoglycan layer. Nisin (US Food and Drug Agency approved bacteriocin) produced by *L. lactis* subsp. *lactis* marketed as Nisaplin by Danisco has found to have inhibitory actions against foodborne enteropathogens including *Clostridium difficile* [143, 144]. Similarly, MicroGARD is another FDA approved commercial preparations by Danisco (skim milk fermentate of *Propionibacterium freudenreichii* subsp. *Shermanii*) used as an eminent bio-preservative in various dairy and food matrices [145]. Bacteriocins of LAB not only have their potential applications in food preservations but also in the clinical sector as they revealed the inhibitory potential against various urogenital and antibiotic-resistant pathogens [146, 147].

### Short chain fatty acids

The dietary carbohydrate in the food gets digested by the action of various enzymatic actions and absorbed in the intestine. The food contains not only digestible carbohydrates but also the non-digestible fibers which have got a crucial role to play in human health and nutrition such as providing bulkiness to food, assisting the smooth passage of food in GIT, prebiotics action, and so on. Prebiotics are defined as substrates that are selectively utilized by host microorganisms conferring a health benefit [148]. These non-digestible carbohydrates (prebiotics) are selectively get fermented by commensals and probiotic bacteria in the gut to produces various end products such as carbon-di-oxide, hydrogen, methane, and short-chain fatty acids (SCFAs), primarily acetate, propionate, and butyrate. Lactobacilli synthesize SCFAs from (i) fermentation of carbohydrates to produce as pyruvate by glycolytic pathway (ii) phosphoketolase pathway for heterofermentative bacteria. However, bifidobacteria use fermentation (Bifidus) pathway to produce majorly the acetate and formate under carbohydrates limited condition, whereas, the acetate and lactate during the existence of carbohydrates in excess. The metabolic fate of acetate is that it enters the peripheral circulation and later metabolized by muscles and other tissues, while the liver takes up leftover propionate [149]. By contrast, the butyrate acts as the primary energy source (70% of their energy) of colonocytes (monocarboxylate transporter-1 pathway) and also regulates the colonic microbiome, cellular apoptosis, proliferation, and differentiation of the gut enterocytes [150]. It was earlier investigated that the lower concentrations (< 0.5 mM) of butyrate acts as the energy source to the cells (constructive manner), however, in contrast, higher concentrations of butyrate (0.5–5 mM) was found to inhibit histone deacetylase (HDACi) and arrest the cell cycle with apoptosis by p53-dependent and -independent manner (destructive manner) [151]. Although few studies indicate probiotic LAB does not primarily produce butyrate [152], there have been sufficient findings that showcases the supplementation of probiotics cocktail has significantly enhanced the propionate and butyrate by modulating the gut microbiota [153]. For example, the consortium of acetate, propionate, and butyrate was found effective against the growth of gastro-intestinal pathogens like *Clostridium difficile* (involved in antibiotic-associated diarrhea) and *E. coli* [154]. The extraction and identification of SCFAs rely on solvent extraction and HPLC techniques. Nevertheless, the use of GC–MS is more analytical rather preparative. However, GC–MS provides overall insights on fatty acids in the mixture [153, 155].

In recent days, there has been increasing evidence on the therapeutic approaches of short-chain fatty acids in the management of IBD and colorectal cancer due to their potentiality to overcome the inflammation and proliferation of cancerous cells respectively [156]. The findings of a randomized clinical trial by Cremon et al. indicate a considerable increase in the SCFAs viz. acetate and butyrate and reduction in the pro-inflammatory cytokine IL-15 upon supplementation of *L. paracasei* CNCM I-1572 to the irritable bowel syndrome (IBS) patients [157]. Additionally, SCFAs modulate the Caco-2 trans permeability by enhancing TEER values and tight junction proteins genes expression [158–162], thus suggested the possible influence of SCFAs in modulating the intestinal barrier property and may be relevant for segmented or targeted consumers of different phases of life.

### Vitamins

Vitamins are the organic molecules that are supplemented in the diet in a small amount to facilitate various biological processes in the body. Most B-complex group vitamins are directly involved as coenzymes in several energy metabolism reactions [162]. In contrast, Vitamin K is the only fat-soluble vitamin that acts as a co-enzyme. Humans are incapable of biosynthesizing most of the vitamins, and therefore they subsequently have to be supplemented exogenously. Most of the vitamins have to be supplemented through the diet (vitamin A, D, E, etc.), however, limited vitamins (folic acid-B9, cobalamin-B12, Riboflavin-B2) are even synthesized by commensal gut bacteria and some probiotic bacteria [163]. Although most of the vitamins exist in natural food systems, vitamin deficiency is still a significant challenge for the medical fraternity, majorly due to the malnutrition, unbalanced diets, and altered food habits. B-group vitamins, normally present in many foods, are easily destroyed during the thermal processing of foods. For this reason, the fortification of certain foods with specific vitamins is necessary. In contrast, the dietary intervention of various in situ vitamins producing LAB (Table 2) is also a benign approach to overcome such deficiency. The use of such microorganisms is also an economically viable alternative than fortification with chemically synthesized pseudo-vitamins. This indeed allows the production of foods with enhanced levels of vitamins that are rare to cause side effects. Cobalamin is most preferably produced by industrial microbial fermentation, as chemical synthesis is very costly. However, cheap agro-byproducts are most preferable as a raw material for such fermentations. In this connection, Deptula et al. utilized a whey-based liquid medium for the production of vitamin B12 from *Propionibacterium freudenreichii* 2067 [164]. The identification of biosynthesized vitamins from natural or over-expressed LAB strains can assess by spectrophotometrically and chromatographic (HPLC) techniques depending on the type of vitamins [164, 165].

**Table 2:** Vitamins and short chain fatty acids producing LAB and other adjunct cultures

Organisms	Components	References
*Streptococcus gallolyticus* subsp. *macedonicus* (*S. macedonicus*) CRL415	Folate	[203]
*Propionibacterium freudenreichii* DSM 20271	Vitamin B12	[204]
*E. faecalis*, *L. helveticus,* and *L. acidophilus*	Formic acid, acetic acid, vitamin B1	[205]
*L. brevis*, *L. plantarum*, and *L.pentosus*	Acetic, propionic butyric, isobutyric isovaleric	[206]
*Propionibacterium freudenreichii*	Vitamin B12	[207]
*L. pentosus* var. *plantarum* BFP32	Acetic, butyric, and propionic acid	[208]
*L. pentosus* var. *plantarum* BFP32	Vitamins B1 and B2	[208]
*Lactobacillus* spp.	Vitamin B12	[209]
*L. sakei*, *L. plantarum*	Folate	[210]
*L. plantarum* and *L. coryniformis*	Vitamin B12	[210]
*L. delbrueckii* subsp. *bulgaricus* CRL 863, *S. thermophilus* CRL 415, and CRL 803	Folate	[203]
*Propionibacterium freudenreichii* DF15	Vitamin B12	[211]
*L. plantarum*, *L. reuteri*, *L. brevis*, and *L. fermentum*	Folate	[211]
*B. breve* M-16 V	Acetate	[212]

The genome annotation of 256 human gut bacteria revealed the enzymes for biosynthesis pathways for eight B-vitamins (biotin, cobalamin, folate, niacin, pantothenate, pyridoxine, riboflavin, and thiamin) [166]. Amongst LAB, lactococci and *Lactobacillus* (*brevis*, *fermentum*, *reuteri*, *salivarius*) displayed the complete genes (*ribA*, *ribB*, *ribG*, and *ribH*) for riboflavin synthesis [167, 168]. In a clinical trial, the supplementation of probiotic strain *B. animalis* subsp. *lactis* HNO19 (DR10**™)** among pregnant women resulted in a significant increase in vitamin B6 in the blood concentration and vitamin B12 in the second and third trimester [169]. Such evidence suggests the significance of fermented products in overcoming micronutrients (vitamins) deficiency.

## Concluding remarks

To summarize, ‘paraprobiotics’ (dead/inactive cells of probiotics) and ‘postbiotics’ (healthful metabolites of probiotics) are the evolving concepts in the functional biotics arena. These have several advantages over the traditional probiotics like known molecular structure, use in purified forms, the specific mechanism of action, better accessibility of MAMP-PRR interaction in triggering a specific downstream pathway, better availability of production process for industrial scale-up, ease in production and storage, etc. The various beneficial properties of parprobiotics and postbiotics include antiinflammatory, gut barrier property, anti-adhesion, anti-biofilm, anti-viral, immunomodulatory, antihypertensive, hypocholesterolemic, anti-proliferative, antioxidant, etc. attributes have documented yet. These attributes suggest the potentiality of paraprobiotic and postbiotic molecules to enhance the host health by modulating the host physiology (ameliorating the disease condition or preventing the onset of disease condition). But, there is a high need for human/clinical trials focusing on the validation of health claims of these bioactive molecules. The trials in immunocompromised subjects would be further augmentable to investigate the tolerance of immunocompromised subjects on these biomolecules. On the other hand, we do lack of knowledge about the stability of paraprobiotics and postbiotics under in vitro and in vivo digestive conditions to comprehend specific mechanistic actions by interacting with the ligands.
